# Rituximab in the Management of Refractory Myasthenia Gravis and Variability of Its Efficacy in Anti-MuSK Positive and Anti-AChR Positive Myasthenia Gravis

**DOI:** 10.7759/cureus.19416

**Published:** 2021-11-09

**Authors:** Sanjiv Bastakoti, Saru Kunwar, Sujan Poudel, Jonathan Quinonez, Seema Bista, Navpreet Singh, Vivek Jha, Samir Ruxmohan, Sylvia Paesani, Wilson Cueva, Jack Michel

**Affiliations:** 1 Division of Research & Acadamic Affairs, Larkin Community Hospital, South Miami, USA; 2 Internal Medicine, KIST Medical College & Teaching Hospital, Kathmandu, NPL; 3 Intensive Care Unit, Metrocity Hospital and Research Center, Pokhara, NPL; 4 Neurology/Osteopathic Neuromuscular Medicine, Larkin Community Hospital, South Miami, USA; 5 Neurology, Larkin Community Hospital, South Miami, USA; 6 Family Medicine, Larkin Community Hospital, South Miami, USA; 7 Internal Medicine, Larkin Health System, South Miami, USA

**Keywords:** anti-achr antibody, rituximab, refractory myasthenia, myasthenia gravis, monoclonal antibody

## Abstract

Myasthenia gravis affects the neuromuscular junction of the skeletal muscles. It results in muscle weakness involving skeletal muscles (diaphragm, extraocular muscles) and myasthenic crisis. Treatment options for myasthenia gravis management have expanded, including azathioprine, corticosteroids, plasma exchange, and tacrolimus. Unfortunately, a few cases of myasthenia gravis don't respond to conventional treatment modalities. Monoclonal antibodies, rituximab (RTX), are novel treatments that have garnered interest as of late due to their efficacy within the patient population presented with refractory form myasthenia gravis. This review aims to showcase how RTX is an effective treatment within different forms of myasthenia gravis. A limited review was performed using databases that include PubMed and Google Scholar. The following keywords were used: "myasthenia gravis," "rituximab," "monoclonal antibody," "anti-AChR antibody," and "refractory myasthenia." The review focused on case reports, human studies, or research surveys based on the inclusion criteria of human studies involving participants more than 18 years of age and published in English literature. Out of 69 articles, 14 were duplicates, and 29 were relevant and met the inclusion criteria. The findings from the study demonstrate that patients with refractory myasthenia gravis responded well to RTX treatment. Furthermore, RTX has been shown to decrease corticosteroid dependence, induce sustained remission, and have a favorable response to anti-MuSK antibody positive myasthenia gravis compared to anti-AChR antibody positive myasthenia gravis. This literature review suggests that patients with refractory myasthenia gravis can benefit from rituximab; however, it has a variable response in different forms of myasthenia gravis.

## Introduction and background

Myasthenia gravis (MG) is an autoimmune disorder characterized by muscle weakness that worsens with exertion and improves with rest. Extrinsic ocular muscle (EOM) weakness is the initial symptom in two-thirds of patients, which progresses to involve bulbar muscles and limb muscles and later results in generalized MG. It is a rare disease with a prevalence of 20 per 100,000 individuals in the United States population [1]. However, the prevalence of treatment-refractory MG increased tenfold, from one per 200,000 to one per 17,000 from 1930 to 1955 [2]. MG is predominantly caused by autoantibodies against skeletal muscles' nicotinic acetylcholine receptor (AChR) [3]. But around 10-20% of patients with myasthenia gravis are found to be seronegative for AChR antibodies, and antibodies to the muscle-specific tyrosine kinase (MuSK) are found in 0-70% of MG patients [4].

For myasthenia gravis, wide groups of treatment options are available, ranging from combination treatment with acetylcholinesterase inhibitors, immunosuppressant, and thymectomy in particular groups of patients [5]. Corticosteroids, thymectomy, and azathioprine are the initial immunosuppressive treatment options for myasthenia gravis [6]. In addition, plasmapheresis and intravenous immunoglobulin (IVIg) are required in severe cases [7]. Even though around 80-85% of patients respond well with available treatment options, around 15% are refractory to conventional treatment options and require alternative forms of treatment such as rituximab (RTX) and cyclophosphamide eculizumab, and other novel immunomodulatory therapies [8]. The therapeutic options like corticosteroids and other immunosuppressive medications can be complicated by several adverse effects related to the prolonged use of drugs which ultimately leads to a decrease in the quality of life in patients with myasthenia gravis. Furthermore, the availability of medications like RTX and other novel immunomodulatory therapies, their role in inducing disease remission and sustaining the remission has broadened the horizon for treatment of myasthenia gravis. 

RTX is a genetically engineered chimeric monoclonal anti-CD20 antibody that acts mainly by antibody-dependent cytotoxicity, complement-dependent cytotoxicity, and induction of apoptosis of CD20+ cells resulting in the elimination of B cells (normal and abnormal) from the body and allowing new B cells to develop [5,9]. In addition, RTX is also known to have a steroid-sparing effect in anti-MuSK antibody-positive MG [8]. This paper aims to determine the challenges and opportunities in the diagnosis of myasthenia gravis, to showcase the role of RTX in refractory generalized MG, to elucidate the differential response of RTX in MuSK antibody-positive MG as compared to AChR antibody-positive MG, and to determine the role of RTX in inducing sustained remission in patients with refractory myasthenia gravis.

Forty-nine articles were selected after a thorough screening process using titles, abstract, and full-text articles. The following keywords were used: "rituximab," "myasthenia gravis," "monoclonal antibody," "anti-AChR antibody," and "refractory myasthenia." Out of 49 articles from the PubMed and Google Scholar databases, 14 articles were removed for duplicates, and 29 articles were relevant and met the inclusion criteria for this review paper. The articles published between 2011 and 2020, written in English, and with a study population of adults older than 18 years, are included in this study.

## Review

Challenges and opportunities in the diagnosis of myasthenia gravis

For the appropriate treatment of patients, there has to be a consistent definition of 'refractory myasthenia gravis (RMG),' but currently, there is no evidence-based and widely accepted definition available. Different clinical trials and literature have used different criteria to define RMG, making it difficult to compare study results and derive conclusions for the management of individual patients. A retrospective study in 2016 done by Sudulagunta et al. defined RMG as those who could not be lowered on immunotherapy without clinical relapse, those who are not clinically controlled on immunotherapy regimen, or who had developed severe side effects from immunosuppressant therapy for at least 12 months [10]. A similar retrospective study done by Collongues in 2012 characterized RMG as patients with MG who failed to respond to thymectomy and had at least one to two successive immunosuppressive drugs with or without associated oral corticosteroids [11]. In addition to these criteria, previously used definitions include repeat hospitalization or regularly planned hospitalizations and deterioration lasting < 24 months. Older definitions also do not consider the improved outcomes achieved with IVIg, plasma exchange, and newer drugs.

Considering the various definitions used in different studies, Mantegazza and Antozzi summarized RMG as described in Table 1 [2].

**Table 1:** Commonly Used Criteria to Define Refractory Myasthenia Gravis (Adapted From Mantegazza and Antozzi, 2018) [2] RMG - Refractory Myasthenia Gravis, IVIg- Intravenous Immunoglobulin, PLEX - Plasma Exchange, MG - Myasthenia Gravis

No.	Criteria	Description
1	Failure to respond adequately to conventional therapies OR	Persistent moderate to severe weakness, i.e., insufficient response to the maximum safe doses of corticosteroids and adequate dose and duration of at least one immunosuppressant
2	Reduction of immunosuppressive therapy not possible without clinical relapse or ongoing rescue therapy needed like IVIg or PLEX OR	Although patients may initially respond to immunosuppressive therapy because of the potential side effects associated with their prolonged use, the duration of such therapies must be restricted
3	Severe or intractable adverse effects from immunosuppressive therapy	Described more accurately as 'treatment intolerant' however, the inability to effectively treat MG using conventional immunosuppressive agents has the same result as being treatment-refractory, hence frequently used as a defining criteria

Role of rituximab in refractory generalized myasthenia gravis

**Figure 1:**
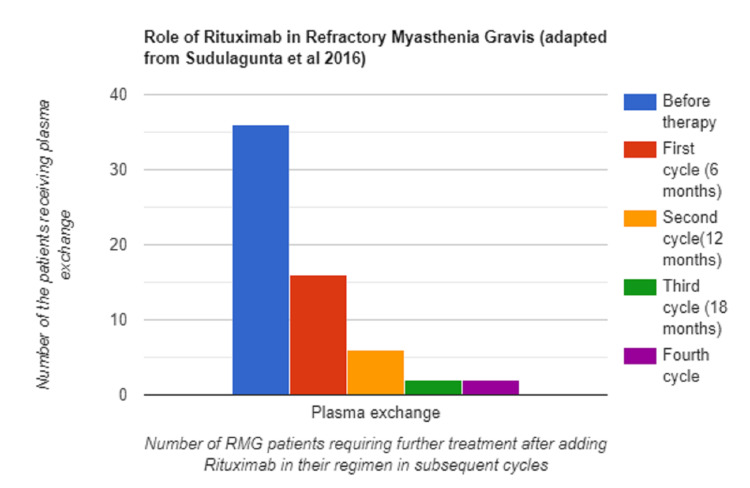
Role of Rituximab in Refractory Myasthenia Gravis (Bar Diagram Adapted From a Study by Sudulagunta et al. in 2016) [10] RMG - Refractory Myasthenia Gravis

The efficacy of RTX has been evaluated in several case reports and retrospective case series [10,12-14]. The diagram below represents the findings from the retrospective study done by Sudulagunta et al. in 2016 in 76 RMG patients (Figure 1) [10]. Among the 42 RMG patients, 39 patients received treatment with prednisolone, 36 patients received plasma exchange, and three received mycophenolate mofetil [10]. The dose of prednisolone could be reduced in patients after treatment with RTX, and after the end of the second cycle at 12 months, 15 patients were tapered off from prednisolone [10]. There was a reduction in the number of patients receiving plasma exchange after treatment with subsequent cycles of RTX (Figure 1) [10]. In addition to this, the study also concluded that there was a significant decrease in the dosage requirement of mycophenolate mofetil and a decrease in AChR antibody titers after adding RTX to their therapy [10].

**Figure 2:**
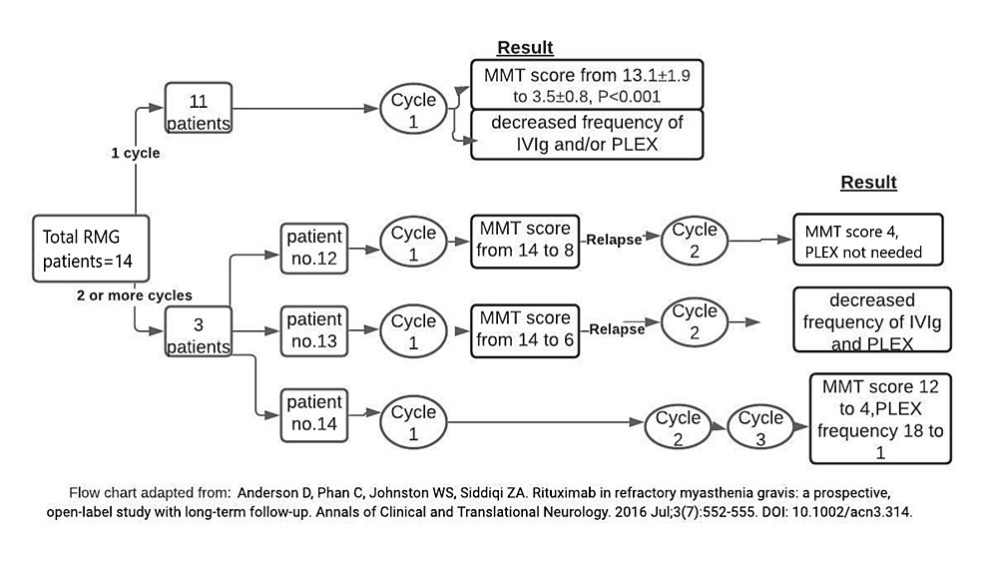
Role of Rituximab in Refractory Myasthenia Gravis (Adapted From Anderson et al., 2016) [15] MMT- Manual Muscle Testing, PLEX - Plasma Exchange, RMG - Refractory Myasthenia Gravis

Anderson et al. performed a prospective open-label study at the University of Alberta from 2012 to 2016 [15]. There were a total of 14 RMG patients, and they were given RTX every week at the dose of 375 mg/m^2^ for four weeks and then every month for two months or at the dose of 750 mg/m^2^ for one month every two weeks [15]. They were then followed up for approximately two years [15]. The results are summarized in the flow diagram below (Figure 2) [15].

Another retrospective multicenter study done by Nicolas Collongues and his colleagues in 2012 involved 13 RMG patients and found that after induction with RTX, the annualized relapse rate (ARR) decreased from 2.1 to 0.3 (p < 0.001), and Myasthenia Gravis Foundation of America scores also decreased from 5-3b to 4b-0 [11]. Corticosteroids were also successfully tapered off in seven RMG patients [11]. A meta-analysis by Chu et al. provided more detailed information on corticosteroid dose reduction after the addition of RTX to the therapy [12]. They found that the dose was reduced by 21.70 (15.50, 42.46) mg/d (before treatment 31.81 mg/d and after treatment 6.81 mg/d; Z = 2.366, p = 0.018) [12].

An 18-month follow-up study in eight RMG patients in India also demonstrated the efficacy of RTX as induction therapy [13]. Four cycles of RTX were given, and there was a significant reduction in doses of azathioprine and oral corticosteroids in all of the RMG patients, and seven out of eight were successfully tapered off with prednisolone [13]. Regarding repeated RTX infusions, this study concluded that it might not be necessary, especially in low-income countries like India, considering the cost of therapy [13]. In a prospective study by Beecher et al., 22 patients with refractory MG who were treated with RTX reported significant reductions in manual muscle testing (MMT) scores (p < 0.0001) [14]. Sustained clinical improvement was associated with the first cycle of RTX [14]. In 14 patients taking prednisolone, the dose was significantly reduced after RTX therapy (p = 0.002); however, 10 patients had relapses requiring additional cycles of RTX [14].

A retrospective study by Afanasiev et al. found RTX to work in only 50% of patients [16]. But consistent with the prior studies, their study also confirmed that RTX favors a reduction in prednisolone dose. They evaluated 28 RMG patients in their study based on post-intervention status (PIS) every six months from 2004 to 2015. The percentage of patients showing improvement based on PIS were as follows: at M6, 43%; M12: 50%; M18: 39%; M24: 42%; M30: 38.5%; M36: 50%, and hence overall, 50% of patients showed improvement in their study [16]. However, a shortcoming of their study was the delayed use of RTX (average 11.3 years after the onset of MG) as Iorio et al. in their meta-analysis had found that the shorter the duration of the disease before administering RTX, the better was the response [17]. Nevertheless, comparing and contrasting the above findings, RTX appears to be efficient in treating RMG.

Results from the various studies demonstrate that rituximab brings positive clinical outcomes in patients with RMG, as well as it helps in tapering the dose of the traditional immunosuppressant drugs, thus reducing adverse effects related to these drugs. RTX was effective as induction therapy, and it was also effective in sustaining remission in patients with RMG. In developing countries like India, a study has demonstrated that RTX is an effective agent for induction of remission in RMG patients; however, the study's sample size was small [13]. A study by Sudulagunta et al. demonstrated that the sessions of plasma exchange were reduced after treatment with rituximab [10]. All the above-mentioned favorable outcomes of rituximab in refractory myasthenia gravis patients have widened the options for treatment of refractory myasthenia gravis; they have also demonstrated that RTX could improve patients' quality of life by decreasing dose-related adverse effects and sustaining remission [10,12-17]. Even though RMG patients benefit from rituximab, more studies are required to identify those groups of patients who can benefit maximally after treatment with rituximab.

Role of rituximab in MuSK antibody positive myasthenia gravis

An estimated 7% of all patients with MG are MuSK antibody positive. MuSK is a transmembrane receptor tyrosine kinase located in the postsynaptic membrane [18]. MuSK MG is an acute onset disease with rapid worsening of signs and symptoms over a few weeks [18].

In a retrospective study carried out in Austria by Topakian et al. in 56 patients, remission after treatment with RTX was more frequent in those patients with MuSK antibodies than those with AChR antibodies (71.4% vs. 35.9%, p = 0.022) [19]. Out of the 56 patients in the study, 69.6% were MuSK antibody positive, whereas 25% were AChR antibody positive. Before treatment with RTX, these patients were treated with sessions of IVIg and plasma exchange (PLEX). After the last follow-up at the median of 23 months, remission was present in 42.9% of the patients, and 25% had minimal manifestations [19]. In a multicenter, blinded prospective review by Hehir et al., 58% of MuSK MG patients treated with RTX achieved primary outcome compared to 16% of the control group (p = 0.02) [20]. The same study also revealed a steroid-sparing effect of RTX, where among the patients with MuSK MG who were pretreated with RTX, only 29% were taking prednisolone compared to 74% of the patients who were taking prednisolone in the control group who were not pretreated with RTX (p = 0.001 and 0.005) [20]. In addition, this steroid-sparing effect of RTX is further highlighted by a study where the dose of prednisolone decreased by 65.1%, 85.7%, and 93.8% after one, two, and three cycles of RTX therapy, respectively [21]. Tandan et al. studied the safety and efficacy of RTX among 169 patients with MG [22]. Modified Myasthenia Gravis Foundation of America post-intervention scale of minimal manifestations (MM) occurred in 44% and combined pharmacological and chronic stable remission in 27% [22]. MM was achieved in 72% of MuSK MG and 30% of AChR MG (p < 0.001) [22]. Moreover, post-treatment relapses decreased more in MuSK MG (p = 0.05). After treatment with RTX, 26% of AChR MG and 82% of MuSK MG patients showed decreased antibody titers [22]. Favorable outcomes to treatment were reported in MuSK MG patients of young age and with less severe disease [22]. In a meta-analysis by Chu et al., the overall rate of RTX treating refractory AChR antibody-positive MG was 77% (p = 0.030) compared to the rate of RTX treating refractory MuSK antibody-positive MG to be 73% (p = 0.048) [12]. The side effects of RTX were infectious pneumonia, leukopenia, paroxysmal atrial fibrillation, and progressive multifocal leukoencephalopathy [12].

Diaz-Manera et al. compared the response of RTX in AChR antibody-positive MG versus MuSK antibody-positive MG in a total of 17 patients [23]. After the post-treatment period of 31 months, six AChR positive patients required reinfusion, but all MuSK MG patients either achieved remission or showed minimal manifestations and did not require reinfusion. Therefore, in MuSK MG, the steroid dose could be tapered, and immunosuppressant could be withdrawn. Clinical outcomes were associated with markedly reduced antibody titers only in the MuSK antibody-positive group of patients. Out of six MuSK antibody-positive patients, three patients were identified as MuSK antibody-negative at the end of follow-up. In addition, three patients showed an 80% reduction in titers of MuSK antibodies [23].

A study by Topakian et al. revealed an association between the presence of MuSK antibodies and remission after treatment with RTX [19]. Beneficial effects of RTX in patients with MuSK antibodies were also revealed by Hehir et al., where more patients with MuSK MG achieved primary outcomes as compared to the control groups [20]. In addition, the study also revealed steroid-sparing of RTX where patients could be tapered from steroids after the initiation of RTX [20]. Tandan et al. also showed that favorable outcomes in MM were present more in MuSK MG than AChR MG [22]. In addition, the study also showed a decreased relapse rate among the MuSK MG group of patients [22]. However, in the meta-analysis by Chu et al., the rate of treatment of refractory MG was more in AChR MG than MuSK MG [12]. Although the studies mentioned above demonstrate that MuSK antibody-positive MG and AChR antibody-positive MG have a variable response to rituximab, further studies are required to compare the efficacy and safety of RTX and relapse rate after treatment with RTX in MuSK MG and AChR MG. Studies showing an association between the favorable outcomes after treatment with RTX and MuSK MG are described below (Table 2).

**Table 2:** Overview of the Studies That Show an Association Between Favorable Outcomes After Treatment With Rituximab and MuSK Antibody-Positive Myasthenia Gravis AChR - Acetylcholine Receptor, MG - Myasthenia Gravis, MM - Minimal Manifestations, MuSK - Muscle-Specific Tyrosine Kinase, RTX - Rituximab

Reference	Study Design	Study Aim	Sample Size	Results
Topakian et al. [19] (Austria)	Retrospective study	To study the use, safety, and efficacy of adult patients with MG	56 patients	Remission was achieved in 71.4% of the MuSK antibody-positive MG vs. 35.9% of the AChR antibody-positive patients (p = 0.022). An outcome of MM was achieved in 85.7% of the MuSK antibody-positive patients and 64.1% of the AChR antibody-positive patients
Chu et al. [12] (China)	Meta-analysis	To evaluate the safety and efficacy of RTX in	A total of 1772 articles, 10 retrospective case analyses, and one observational study among 160 patients taking RTX	The overall rate of RTX treating refractory AChR antibody-positive MG was 77% (p = 0.030) compared with the rate of RTX treating refractory MuSK antibody-positive MG at 73% (p = 0.048)
Manera et al. [23]	Prospective study	To report the immunologic and clinical long-term follow-up of 17 patients with MG and compare the response between AChR positive MG and MuSK positive MG patients.	A total of 17 patients out of which 6 were MuSK positive MG and the remaining 11 were AChR positive MG	It was observed that after a mean follow-up period of 35 months, all of the patients with MuSK positive MG achieved remission or showed minimal manifestations and did not require reinfusion. On the other hand, 6 AChR MG required reinfusions, and they improved, but none of them achieved remission or showed minimal manifestations.
Tandan et al. [22]	Literature review	To explore the efficacy and safety of RTX in patients with MG from case reports and case series	A total of 47 publications, out of which 28 were case reports, 19 were case series consisting of 2-22 patients.	MM was achieved in 72% of MuSK MG and 30% of AChR MG (p < 0.001). Post-treatment relapses decreased more in MuSK MG (p = 0.05). In addition, 26% of AchR and 82% of MuSK MG patients showed decreased antibody titers after treatment with RTX.
Hehir et al. [20]	Multicenter, blinded prospective review	To evaluate the efficacy of RTX in MuSK MG.	55 patients with MuSK MG out of which 24 patients were treated with RTX and 31 patients were the control group	At the end of follow-up, 58% of MuSK MG patients treated with RTX achieved primary outcomes compared to 16% of the control group (p = 0.02). In addition, 29% of the RTX-treated patients took steroids with an average dose of 4.5 mg/day compared to 74% of the controls with a mean dose of 13 mg per day (p = 0.001 and 0.005).

Rituximab in long term remission of refractory myasthenia gravis

Most immunosuppressants used in MG have a delayed onset of action, and they are not sufficient to induce a stable remission; RTX might be necessary for patients who do not respond well with standardized immunosuppressive therapy [17]. A systematic review by Iorio et al. reported a response rate of 83.9% using RTX in a patient group where the majority of the patients had refractory MG [17]. A retrospective study by Afanasiev et al. in 28 patients with refractory MG treated with RTX reported a significant improvement in myasthenic muscle score (MMS) (p < 0.0001) at six months of follow-up [16]. Persistently stable improvement remained for 36 months [16]. Furthermore, the dose of prednisolone was significantly reduced after treatment with RTX [16]. However, 39% of the patients reported side effects, out of which 13% had severe side effects [16]. In a prospective study by Jing et al. in eight patients with refractory generalized MG treated with RTX, there were statistically significant improvements in MMT scores and MG-related activity of daily living (MG-ADL) at three and six months post-treatment with RTX; however, statistically, significant improvements were not found concerning MG-related quality of life [24]. All patients had a reduction in the dose of prednisolone, and at six months follow-up, the mean reduction in the dose of prednisolone was 43% (p = 0.018) [24]. In a retrospective study by Robeson et al., sixteen patients with refractory AChR MG were included, and the effectiveness of RTX was studied [25]. After completing the initial cycle of RTX, ten patients achieved stable remission, three patients achieved pharmacological remission (required either prednisolone or azathioprine), and three patients reported an MM score of zero [25]. A retrospective study by Stieglbauer et al. of four patients with refractory MG showed that all the patients achieved sustained remission and did not require other forms of treatments (steroids and immunosuppressants) after treatment with RTX [26]. In a study conducted by Landon-Cardinal et al., 12 patients were enrolled, and eleven patients completed the study [27]. Although the primary outcome of an improvement of muscle function based on myasthenic muscle score (MMS) at 12 months of follow-up was present in one patient, a favorable outcomes RTX on muscle function was observed in half of the patients at 12 months and persisted in one-third of patients at 18 months [27].

In a retrospective study from the Yale University School of Medicine by Nowak et al., 14 patients with refractory generalized myasthenia gravis were included, consisting of the AChR antibody-positive patient group and MuSK antibody-positive group [21]. PLEX sessions before and after the treatment with RTX were compared in 12 patients [21]. Nine patients did not require PLEX at six months (cycle 1), whereas eleven patients were free of PLEX at 12 months (cycle 2) after the initiation of RTX [21]. Although PLEX was continued after the first cycle in three patients, they did not require PLEX after the third cycle of RTX [21]. One patient was on mycophenolate mofetil at the time of initiation of RTX, and he was able to discontinue mycophenolate mofetil after the first cycle of RTX [21]. In addition, four patients were on azathioprine before the initiation of RTX, and they were AChR positive, and one amongst the four patients was able to discontinue the treatment after the first cycle of RTX [21]. Another patient was able to discontinue azathioprine after the second cycle of RTX [21]. AChR antibody titer was measured before and after the treatment with RTX, which reduced by an average of 40.2%, 52.1%, and 67% after the first, second, and third cycle of RTX, respectively [21]. A retrospective cohort study from the Karolinska University Hospital, Stockholm, by Brauner et al. included 72 patients with MG initiating treatment with low dose RTX or conventional immunosuppressant [28]. Out of the 72 patients, 24 patients had received RTX within the 12 months of the onset of disease (new-onset group), and 48 patients received RTX at a later time, 34 of whom had therapy-refractory disease receiving RTX 12 months or more after the onset of disease and after treatment with at least one conventional immunosuppressant (refractory RTX group). The control group consisted of 26 patients receiving conventional immunotherapies. It was observed that time to remission was shorter in the new-onset group compared to therapy refractory patients (seven vs. 16 months; p = 0.09) [28]. In addition, the time for remission was shorter in the new-onset group than in the control group (seven vs. 11 months; p = 0.04) [28]. Fewer rescue therapies were required in RTX-treated patients compared to the control group in the first 24 months [28]. RTX was associated with more sustained remission than conventional immunotherapies [28]. Furthermore, RTX treated patients tapered off more rapidly from immunomodulatory drugs, including corticosteroids [28]. Finally, RTX was associated with a lower rate of drug discontinuation due to adverse effects than conventional immunotherapies (3% vs. 46%; p < 0.001), indicating better tolerability and adherence to treatment [28].

Studies by Anderson et al. and Nowak et al. revealed sustained remission and improvement in clinical status after treatment with RTX in patients with refractory MG [15,21]. Patients were tapered from the conventional immunotherapies, and some patients who had relapses showed clinical improvement after additional cycles of RTX [15,21]. Jing et al. showed significant clinical improvement after three months and six months post-treatment with RTX in patients with refractory MG [24]. Afanasiev et al. showed sustained improvement after treatment with RTX that lasted up to 36 months [16]. These studies showed the role of rituximab in inducing sustained remission. Comparing the effectiveness of conventional immunotherapies and rituximab is important, and studies have shown that rituximab is superior to conventional immunotherapies in inducing remission. Brauner et al. revealed that as compared to conventional immunotherapies, RTX was associated with more sustained remission [28]. Various factors might affect the induction of remission in patients with MG treated with rituximab. Some patients achieved remission sooner, and Robeson et al. demonstrated that remission was achieved sooner if the treatment with RTX was initiated earlier rather than later [25]. Moreover, Robeson et al. showed stable remission after RTX in patients with AChR antibody MG, but more studies compared rituximab's efficacy in MuSK MG vs. AChR MG are required [25]. Although the studies mentioned above demonstrate that RTX can induce sustained remission in patients with RMG, studies evaluating the effectiveness of RTX over a long duration of time are lacking. Most of the studies evaluated the effectiveness of RTX from one year up to 36 months. Therefore, further studies are needed to assess the long-term efficacy of rituximab. The findings of various studies demonstrating the role of RTX in the sustained remission of patients with MG are summarized (Table 3).

**Table 3:** Various Studies Showing the Role of RTX in Sustained Remission in a Patient With Refractory MG AChR - Acetylcholine Receptor, MG - Myasthenia Gravis, MMS - Myasthenic Muscle Score, MMT- Manual Muscle Testing, RTX- Rituximab

Reference	Study Design	Study Aim	Sample Size	Results
Afanasiev et al. [16]	Retrospective study	To evaluate the efficiency and tolerance of RTXin the management of refractory MG	28 patients with refractory MG	Significant improvement in myasthenic muscle score was observed at six months, and persistently stable improvement remained for 36 months
Jing et al. [24]	Prospective study	To investigate the effect of a low dose of RTX in refractory generalized MG.	Eight patients with refractory generalized myasthenia gravis were treated with RTX	Statistically significant improvements in MMT scores and MG-related activity of daily living (MG-ADL) were observed at three and six months post-treatment with RTX.
Landon-Cardinal et al. [27]	An open-label prospective multicentre pilot phase II trial	To study the efficacy of RTX in refractory generalized MG with AChR antibodies.	12 patients were enrolled, but eleven patients completed the study.	Although the primary outcome was achieved in a single patient at 12 months, the beneficial effect of RTX on muscle function was seen in half of the patients at 12 months and persisted in one-third of patients at 18 months.
Robeson et al. [25]	Retrospective study	To evaluate the durability of response to RTX in the treatment of AChR antibody-positive MG.	16 patients with refractory AChR MG	After completing the initial cycle of RTX, ten patients achieved stable remission, three patients achieved pharmacological remission (required either prednisolone or azathioprine), and three patients reported an MM score of zero
Brauner et al. [28]	A retrospective cohort study	To assess RTX role in refractory and new-onset generalized myasthenia gravis and RTX vs. conventional immunotherapy in new-onset disease.	72 patients with MG initiating treatment with low dose RTX or conventional immunosuppressant and control group of 24 patients receiving conventional immunotherapies	The new-onset group's time to remission was shorter than therapy refractory patients (7 vs. 16 months; p = 0.09). In addition, the time for remission was shorter in the new-onset group than in the control group (7 vs. 11 months p = 0.04). RTX was associated with more sustained remission than the conventional immunotherapies
Nowak et al. [21]	Retrospective study	To study the response of RTX in patients with refractory MG.	14 patients with refractory generalized myasthenia gravis	Sustained clinical improvements were observed in all patients in addition to the reduction of conventional immunotherapies and AChR antibodies titer.
Stieglbauer et al. [26]	Retrospective study	To report on the 10-year outcomes of all four MG patients treated with RTX.	4 patients with refractory MG	All the patients achieved sustained remission and did not require other treatments (steroids and immunosuppressants) after treatment with RTX.

Limitations

This study has several limitations. The application of simple search criteria has limited the number of included articles to only 31. Only two databases were searched, and comprehensive searches of other databases were not done. Any articles which were published in languages other than English literature were excluded. Moreover, most of the studies which were included had small sample sizes. In addition, there are no clear guidelines formulated regarding refractory myasthenia gravis, which has created difficulty in drawing conclusions. Finally, there are no studies present comparing the safety and efficacy of rituximab with newer immunomodulatory drugs.

## Conclusions

This review attempted to determine the roles of rituximab (RTX) on refractory myasthenia gravis (RMG) management. It demonstrates that patients with RMG benefit from RTX use as traditional immunosuppressant treatment can be tapered over time. Furthermore, RTX use can also sustain RMG remission, but it differs between patients with MuSK versus AChR RMG. Overall, RTX is beneficial for RMG, but further studies are needed to determine its variability in efficacy and safety in MuSK antibody-positive MG and AChR antibody-positive MG. More studies are also required to identify the potential patient population group who could benefit from using a novel immunomodulatory drug-like RTX. Future studies may help tailor the treatment options as per patients' requirements and help patients better deal with this cumbersome disease.
